# An extended genovo metagenomic assembler by incorporating paired-end information

**DOI:** 10.7717/peerj.196

**Published:** 2013-10-31

**Authors:** Afiahayati, Kengo Sato, Yasubumi Sakakibara

**Affiliations:** Department of Biosciences and Informatics, Keio University, Hiyoshi, Kohoku-ku, Yokohama, Japan

**Keywords:** Genovo, 454 paired end reads, de novo metagenomic assembler

## Abstract

Metagenomes present assembly challenges, when assembling multiple genomes from mixed reads of multiple species. An assembler for single genomes can’t adapt well when applied in this case. A metagenomic assembler, Genovo, is a de novo assembler for metagenomes under a generative probabilistic model. Genovo assembles all reads without discarding any reads in a preprocessing step, and is therefore able to extract more information from metagenomic data and, in principle, generate better assembly results. Paired end sequencing is currently widely-used yet Genovo was designed for 454 single end reads. In this research, we attempted to extend Genovo by incorporating paired-end information, named Xgenovo, so that it generates higher quality assemblies with paired end reads.

First, we extended Genovo by adding a bonus parameter in the Chinese Restaurant Process used to get prior accounts for the unknown number of genomes in the sample. This bonus parameter intends for a pair of reads to be in the same contig and as an effort to solve chimera contig case. Second, we modified the sampling process of the location of a read in a contig. We used relative distance for the number of trials in the symmetric geometric distribution instead of using distance between the offset and the center of contig used in Genovo. Using this relative distance, a read sampled in the appropriate location has higher probability. Therefore a read will be mapped in the correct location.

Results of extensive experiments on simulated metagenomic datasets from simple to complex with species coverage setting following uniform and lognormal distribution showed that Xgenovo can be superior to the original Genovo and the recently proposed metagenome assembler for 454 reads, MAP. Xgenovo successfully generated longer N50 than Genovo and MAP while maintaining the assembly quality even for very complex metagenomic datasets consisting of 115 species. Xgenovo also demonstrated the potential to decrease the computational cost. This means that our strategy worked well. The software and all simulated datasets are publicly available online at http://xgenovo.dna.bio.keio.ac.jp.

## Introduction

Next generation sequencing (NGS) technologies have allowed an explosion in sequencing with the increased throughput and decrease in cost of sequencing (Scholz, Lo & Chain, 2012). The field of metagenomics has adapted to the new type of sequencing technologies which allows us to generate reads from multiple genomes effectively (Peng et al., 2011). Although a number of metagenomes have been sequenced using NGS, few studies have reported their assembly results (Hiatt et al., 2010; Namiki et al., 2012; Qin et al., 2010). Metagenomes have presented a number of additional assembly challenges, how to assemble multiple genomes from mixed reads of multiple species. In metagenomic data, the number of genomes and the coverage of each genome are initially unknown. The data potentially consists of multiple genomes with inhomogenous coverage distribution (Chen & Pachter, 2005; Lai et al., 2012; Laserson, Jojic & Koller, 2011; Nagarajan & Pop, 2013; Namiki et al., 2012; Peng et al., 2011; Scholz, Lo & Chain, 2012). Assemblers for single genomes can’t adapt well when applied in this case (Lai et al., 2012; Laserson, Jojic & Koller, 2011; Namiki et al., 2012; Peng et al., 2011; Scholz, Lo & Chain, 2012). This assembler generates high rate of misassembled contigs called chimera contig which consists of reads from different species in metagenome assembly (Lai et al., 2012; Mavromatis et al., 2007; Pigmatelli & Moya, 2011).

There are a number of effective assemblers for single genome, but only five attempt to solve metagenome cases: MetaVelvet (Namiki et al., 2012), Meta-IDBA (Peng et al., 2011), IDBA-UD (Peng et al., 2012), MAP (Lai et al., 2012) and Genovo (Laserson, Jojic & Koller, 2011). Metavelvet, Meta-IDBA and IDBA-UD use the De Bruijn graph approach. They were designed to handle short read data. IDBA-UD is an extension of Meta-IDBA solving uneven sequencing depths of different regions of genomes from different species (Peng et al., 2012). MAP was designed for longer reads produced by Sanger (700–1000 bp) and 454 sequencing technology (200–500 bp). MAP uses an improved OLC (Overlap/Layout/Consensus) strategy integrating mate pair information (Lai et al., 2012). Genovo was designed for longer reads of 454 sequencing data; it is a metagenomic assembler under a generative probabilistic model (Laserson, Jojic & Koller, 2011).

Unlike other methods, Genovo assembles all reads without discarding any reads. It doesn’t detect and correct read errors in a preprocessing step. This avoids filtering out any low coverage genomes, hence hopefully is able to extract more information from metagenomic data in order to generate better assembly results. The consequence is high computational cost (Laserson, Jojic & Koller, 2011). Paired end sequencing is currently widely-used yet Genovo was designed for single end reads. In this research, we extend Genovo by incorporating paired-end information, named Xgenovo. We also design algorithms to decrease the computational cost. We modified some procedures of Genovo in determining the location of a read in the coordinate system of contig and offset (the beginning of the read) so that it generates higher quality assemblies with paired end reads. Genovo uses Chinese Restaurant Processes (CRP) to get prior accounts of the unknown number of genomes in the sample. First, we modified CRP by adding a bonus parameter which intends for a pair of reads to be in the same contig also as an effort to solve chimera contig case. Second, we used relative distance for the number of trials in the symmetric geometric distribution instead of using distance between the offset and the center of the contig used in Genovo. For paired end reads, this process should take into account the insert length parameter. Using this relative distance, a read sampled in the appropriate location has higher probability. Therefore a read will be mapped in the correct location.

We used Metasim (Richter et al., 2008) to generate simulated metagenomic datasets. In order to measure the performances more comprehensively, we applied two kinds of species coverage (abundance) distribution for the dataset, uniform and log-normal distribution. In total, we generated 16 simulated datasets from simple to complex datasets. We compared the performance of Xgenovo with the naive use of the original Genovo and the recently proposed matagenome assembler for 454 reads, MAP, which also utilizes paired end information. MAP outperforms standard single genome assemblers for 454 reads, Celera (Myers et al., 2000; Miller et al., 2008) and Newbler (Margulies et al., 2005). In this research, Xgenovo was not compared with single genome and metagenome assemblers which are designed for Illumina types of short read data (<100 bp), like Velvet (Zerbino & Birney, 2008), SOAPdenovo (Li et al., 2010), IDBA (Peng et al., 2010), MetaVelvet (Namiki et al., 2012), Meta-IDBA (Peng et al., 2011) and IDBA-UD (Peng et al., 2012). Xgenovo generated longer N50 than the original Genovo and MAP while maintaining the assembly quality for all datasets. Xgenovo also demonstrated the potential to decrease the computational cost. We successfully extended Genovo by incorporating paired-end information so that it generates higher quality assemblies with paired end reads by modifying Genovo in determining the location of a read in the coordinate system of contig and offset (the beginning of read), different from other assemblers (Koren, Treangen & Pop, 2011; Li et al., 2010; Namiki et al., 2012; Peng et al., 2012; Zerbino & Birney, 2008; Zerbino et al., 2009) which used paired end information to generate scaffolds. The software and all simulated datasets are publicly available online at http://xgenovo.dna.bio.keio.ac.jp.

## Methods

### Overview of Genovo

**Figure 1:**
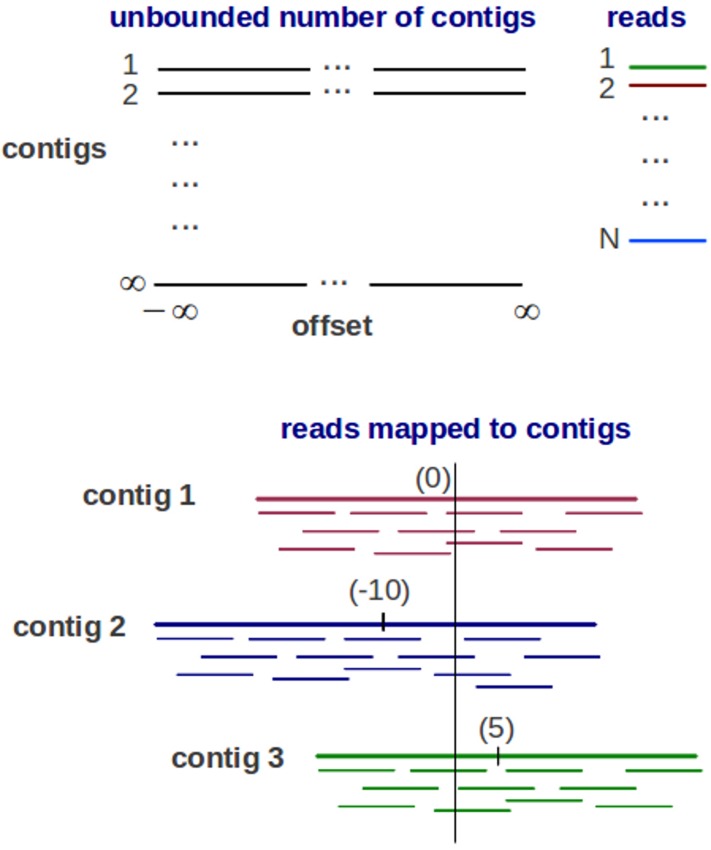
The Generative probabilistic model in Genovo. There are an unbounded number of contigs constructed with unbounded length (from negative infinity to positive infinity) and *N* reads. The reads are mapped to 3 contigs in a coordinate system of contigs and offsets (the beginning location of a read). Each contig has its center. The center of contig 1 is 0, contig 2 is −10 and contig 3 is 5.

Genovo is a metagenomic assembler under a generative probabilistic model, illustrated in Fig. 1. An assembly is represented as a list of contigs and a mapping of each read to the contigs. Each contig is represented as a list of DNA letters {*b_so_*}, where *b_so_* is the letter at position *o* of contig *s*. The mapping represents the position of each read *x_i_* in a coordinate system of contigs and offsets, each read has its contig number *s_i_* and its offset *o_i_* (starting location of the read within the contig). Each read mapped to the contig is aligned base-for-base denoted by *y_i_*. To represent a set of variables, bold-face letters is used, for example, ***b*** represents a set of DNA letters. The probabilistic model is described as below:

1. Construct infinite number of contigs consisting of infinitely many DNA letters. Assume that there are infinitely many contigs consisting of an infinite number of DNA letters sampled following uniform distribution, shown in Fig. 1. Because of the finite number of reads, only a finite number of infinitely many contigs will have reads mapped to them.
2. Map each read to the contigs.

There is a coordinate system of contigs and offsets showing the position of reads mapped to the contigs. Two steps are used in mapping process: first, partition the reads (*N*) to clusters using CRP shown in (1). The number of clusters represents the number of contigs (*s*) as an initial number of multiple genomes. The parameter α controls the expected number of classes.

(1) \begin{eqnarray*} \displaystyle s\sim C R P(\alpha ,N)&&\displaystyle \end{eqnarray*}

Second, assign each cluster of reads to a contig. A good contig is defined as a contig having the most reads towards the center of the contig. Therefore, a starting point of read *o_i_* within each contig is assigned using a symmetric geometric distribution, shown in (2). The parameter ρ_*s*_ controls the length of a contig.

(2) \begin{eqnarray*} \displaystyle {o}_{i}\sim G({\rho }_{s})\quad \forall i=1..N&&\displaystyle \end{eqnarray*}

3. Copy the letters of each read *x_i_* (with some noises) to the mapped location in contigs starting from position *o_i_* with orientation, insertion and deletion encoded by alignment *y*_*i*,_ shown in (3), *l_i_* is the length of *read_i_*, ρ_*ins*_ is the probability of insertion, ρ_*del*_ is the probability of deletion, ρ_*mis*_ is the probability of incorrect copying (mismatch) and *A* is the distribution representing the noise model known for the sequencing technology.

(3) \begin{eqnarray*} \displaystyle {x}_{i},{y}_{i}\sim A({l}_{i},{s}_{i},{o}_{i},b,{\rho }_{\mathit{ins}},{\rho }_{\mathit{del}},{\rho }_{\mathit{mis}})&&\displaystyle \end{eqnarray*}

**Figure 2:**
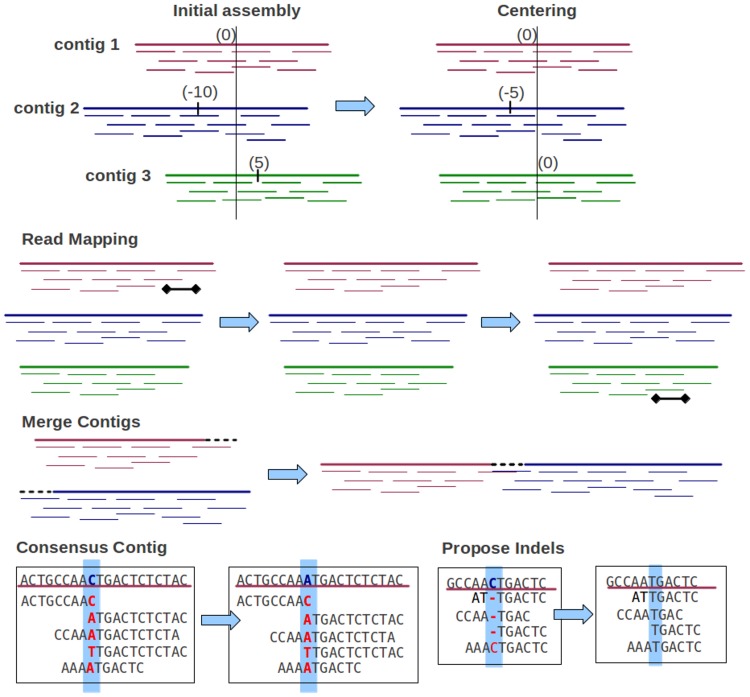
Iterative procedures of Genovo. 5 iterative procedures are illustrated. The centering procedure makes the center of contigs towards zero: 3 contigs with centers (0, −10, 5) become (0, −5, 0) after centering. The read mapping procedure moves reads to the more appropriate location: a read in contig 1 is moved to contig 3. The merge procedure merges two contigs: contig 1 and contig 2 are merged. The consensus contig procedure updates the letter in the consensus contig: letter C in the consensus is changed to A. The propose indels procedure: most reads have an insertion, therefore it is proposed to delete the corresponding letter (C) in the contig and realign the reads.

To generate appropriate assemblies, Genovo performs a series of iterated hill climbing procedures, maximizing or sampling local conditional probabilities to reach MAP solution (the best likelihood), illustrated in Fig. 2. This algorithm is run until convergence (200–300 iterations). Genovo outputs the best assembly, the model with the highest probability during the iterations. The likelihood of this model consists of the likelihood of the alignments log *p*(***x***, ***y***|***s***, ***o***, ***b***), the likelihood for generating (uniformly) each contig letter log *p*(***b***), the likelihood of contigs log *p*(***s***), and the likelihood of offsets log *p*(***o***|***s***, ρ), shown in (4)–(8), where *S* is the number of contigs, *N_s_* is the number of reads in contig *s*, *L* is the total length of all contigs, ρ_*s*_ is the control parameter of the length of a contig, β is the count of DNA character = 4, ${\mathit{score}}_{\mathit{READ}}^{i}$ is the alignment score of *read_i_* mapped to the contig, and

\begin{eqnarray*} \displaystyle {O}_{s}=\sum _{k=1}^{{N}_{s}}\vert {o}_{k}\vert &&\displaystyle \end{eqnarray*}

(4) \begin{eqnarray*} \displaystyle \log \hspace{0.167em} p(x,y\vert s,o,b)+\log \hspace{0.167em} p(b)+\log \hspace{0.167em} p(s)+\log \hspace{0.167em} p(o\vert s,\rho )&&\displaystyle \end{eqnarray*}

(5) \begin{eqnarray*} \displaystyle \log \hspace{0.167em} p(x,y\vert s,o,b)=\sum \mathit{score}_{\mathit{READ}}^{i}&&\displaystyle \end{eqnarray*}

(6) \begin{eqnarray*} \displaystyle \log \hspace{0.167em} p(b)=-\log \vert \beta \vert L&&\displaystyle \end{eqnarray*}

(7) \begin{eqnarray*} \displaystyle \log \hspace{0.167em} p(s)=S\log (\alpha )+\sum _{i=1}^{S}\log \hspace{0.167em} \Gamma ({N}_{s})+\mathit{const}(\alpha ,N)&&\displaystyle \end{eqnarray*}

(8) \begin{eqnarray*} \displaystyle \log \hspace{0.167em} p(o\vert s,{\rho }_{s})=\sum _{i=1}^{S}\left[{O}_{s}\log (1-{\rho }_{s})+{N}_{s}\log \hspace{0.167em} {\rho }_{s}+\mathit{const}(N)\right]&&\displaystyle \end{eqnarray*}

The procedures are described as below:

1. Consensus contig
  This procedure attempts to increase the likelihood of alignment by updating over the DNA letter variable in contigs *b_so_*. The letters of prior contigs are sampled following uniform distribution, therefore the likelihood is maximized by tuning up the number of reads in the current assembly which align the letter *b*∈*B* to the location (*s*, *o*). ${b}_{s o}={\mathit{argmax}}_{b\in B}{a}_{s o}^{b}$ where ${a}_{s o}^{b}$.
2. Read mapping
  This procedure is the main procedure in Genovo: moving reads to the more appropriate location. It performs stochastic ICM updates over the read variables *s_i_*, *o_i_*, *y_i_* sequentially for each read *x_i_*. First, read *x_i_* is removed, then a new location is sampled from the joint posterior *p*(*s_i_* = *s*, *o_i_* = *o*, *y_i_* = *y*|*x_i_*, ***y***_−*i*_, ***s***_−*i*_, ***o***_−*i*_, ***b***, ρ).
3. Global moving
  These procedures change a set of variables at once which speed up convergence. These procedures consist of:
  (a) Propose indels
    If most mapped reads have an insertion at a specific location then the deletion of the corresponding letter in the contig will be proposed and the reads will be realigned. While vice versa, if most mapped reads have a deletion at a specific location, the insertion will be proposed. If the likelihood improves, the proposal will be accepted.
  (b) Centering the contigs
    Each contig has a center. A good contig is defined as a contig having the center towards zero. This procedure shifts the coordinate system of each contig to maximize the likelihood of offset by making the center of the contigs towards zero. In the illustration shown in Fig. 2, there are 3 contigs. After implementing this procedure, the center of each contig shifts towards zero.
  (c) Merge
    It is common for two contigs to have overlapping ends. The assembly created when merging two such contigs would have a higher probability of the model, but if the assembly is only generated by the “read mapping” procedure, it requires multiple iterations. If the end of a contig overlaps with the beginning of another contig, then Genovo will align those ends, the reads in the overlapping area are re-aligned and both contigs are merged. This procedure will be executed if it improves the likelihood of model.
  (d) Chimeric read solving
    Chimeric reads are reads having two segments of length >20 that mapped to noncontiguous areas of the reference genome (Lasken & Stockwell, 2007). The Genovo algorithm assumes that these reads often reach the end of an assembled contig. To solve this case, Genovo disassembles the reads assembled in the end of a contig occasionally (every 5 iterations). Using this procedure, other correct reads or contigs can merge with it and the likelihood of model will increase. If a disassembled read is not chimeric, it will be reassembled appropriately in the next iteration and the likelihood of model will be maintained like the previous iteration.

### Extended Genovo

We extended Genovo by modifying some procedures in order to fit in with paired end reads incorporating paired-end information; this model is called Xgenovo. First, we modified CRP by adding a bonus parameter. Second, we modified the sampling process of the location of a read in a contig. Xgenovo doesn’t use the chimeric read solving procedure from Genovo because it will decrease the likelihood of model. In the extended model, greater numbers of pairs of reads in the contigs increase the likelihood of model. In the chimeric read solving procedure, the reads assembled in the end of a contig disassemble occasionally. The reads may be mates to other reads in a contig, the number of pairs of reads will decrease therefore the likelihood of model also will decrease.

#### Modified CRP

Genovo uses CRP to cluster the reads. The concept of CRP is that the rich get richer. The probability of the new customer sitting at an occupied table is proportional to the number of customers already sitting at it and the probability of the new customer sitting at the next unoccupied table is proportional to a concentration parameter, α, represented by (9). In the assembly case, a customer is a read while a table is a contig. The concentration parameter determines the intention of a new customer sitting at a new table. The customer inclines to sit at the most popular tables (Johnson, 2012). A CRP is a conditional distribution which is invariant to the order of the items (Aldous, 1985) which, in our case, are the reads.

(9) \begin{eqnarray*} \displaystyle p({s}_{i}=s\vert {s}_{-i})\sim \left\{\begin{array}{@{}l@{}} \displaystyle {N}_{-i,s}\quad s\text{: an existing contig}\\ \displaystyle \alpha \quad s\text{: new contig} \end{array}\right.&&\displaystyle \end{eqnarray*}

**Figure 3:**
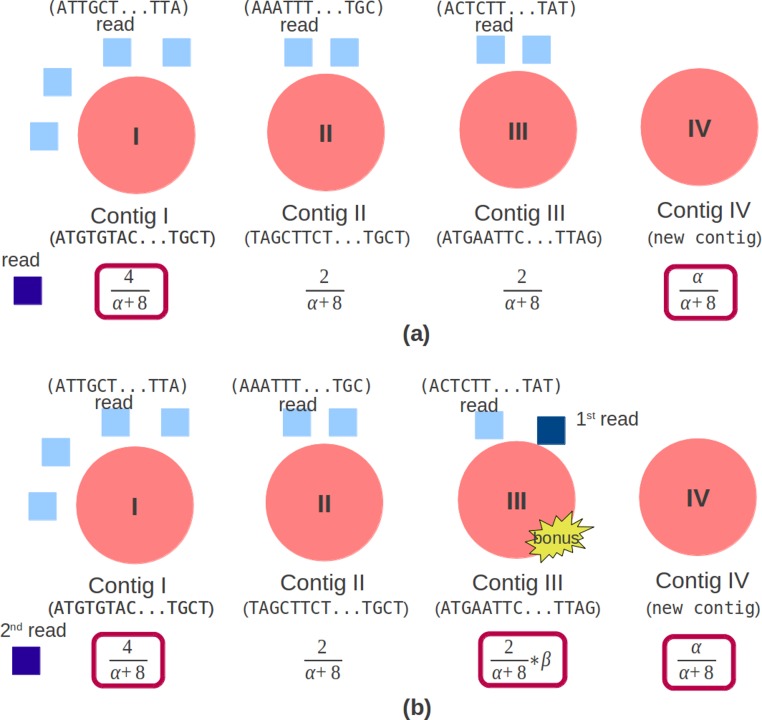
Original and modified CRP. The red circle is a contig, the light blue square is a read mapped in the contig. (A) Original CRP: the dark blue square is a new read which will be mapped. There are 3 contigs (Contig I, Contig II and Contig III) with reads (4, 2, 2) and 1 new contig (Contig IV). (B) Modified CRP: the dark blue square is paired end reads, the 1^st^ read is mapped in Contig III and the 2^nd^ read will be mapped. There are 3 contigs (Contig I, Contig II and Contig III) with reads (4, 2, 2) and 1 new contig (Contig IV). If the 2^nd^ read is mapped in Contig III, a bonus will be given.

*N*_−*i*,*s*_ counts the number of items, not including *i*, that is in contig *s*. For paired end reads, beside being concerned with the concept that the rich get richer, it should also care whether a pair of reads are in the same contig. Therefore, we give a bonus if a read is in the same contig with its mate. In the illustration shown in Fig. 3A, a read chooses a contig in single read case. There are 3 contigs (Contig I, Contig II and Contig III) with reads (4, 2, 2) and a new contig (Contig IV) can be created. The contig which will be chosen depends on the number of reads in the contig and the concentration parameter, α, so that the candidate contigs are contig I (having the most read) and contig IV.

In the paired end read case, illustrated in Fig. 3B, aside from the number of reads in the contig and the concentration parameter, it should also depend on the bonus parameter, represented by (10). This bonus parameter (β) intends for a pair of reads to be in the same contig and as an effort to solve chimera contig case. Therefore the candidate contigs are contig I (having the most reads), contig III (having its mate) and contig IV. If the 2^nd^ read is mapped in Contig III, a bonus will be given.

(10) \begin{eqnarray*} \displaystyle p({s}_{i}=s\vert {s}_{-i})\sim \left\{\begin{array}{@{}l@{}} \displaystyle {N}_{-i,s}\ast \beta \quad s\text{: a mate contig}\\ \displaystyle {N}_{-i,s}\quad s\text{: an existing contig}\\ \displaystyle \alpha \quad s\text{: new contig} \end{array}\right.&&\displaystyle \end{eqnarray*}

#### Modified sampling process of an offset

Sampling process of an offset means assigning a location of the read’s offset at a contig. Geometric distribution represents the probability distribution of the number *y* = *x*−1 of failures before the first success, shown in (11), *p* is the probability on each trial and *k* is the number of trials (Degroot & Schervish, 2011).

(11) \begin{eqnarray*} \displaystyle P(x=k)=(1-p)^{k}p&&\displaystyle \end{eqnarray*}

Genovo uses this concept. Sampling the beginning of a read (an offset) at a location *x* means that Genovo get failures for sampling an offset at location 1 until *x*−1 and success at location *x*. Genovo uses the negative and positive integer for the offsets representation in the contigs. A good contig is defined as a contig having the most reads towards the center of contigs. Therefore Genovo uses a symmetric variation of geometric distribution that includes all the negative integers with a center at 0 to sample a starting point *o_i_* of read within each contig, shown in (12).

(12) \begin{eqnarray*} G(o;{\rho }_{s})=\left\{\begin{array}{@{}l@{}} \displaystyle 0.5(1-{\rho }_{s})^{\vert {o}_{t}\vert }{\rho }_{s}o\not = 0\\ \displaystyle {\rho }_{s}o=0 \end{array}\right. \end{eqnarray*}

$(1+{N}_{s},1+\beta +{O}_{s})=\frac{{N}_{s}}{{N}_{s}+\beta +{O}_{s}}$. The number of trials, |*o_t_*|, is the distance between the offset and the center (the absolute value of the offset). The parameter ρ_*s*_ controls the length of a contig. This parameter is the same with the probability of success on each trial *p* in the original geometric distribution. As the posterior distribution of *p* can be determined if a Beta(α, β) prior is given (Degroot & Schervish, 2011), Genovo also uses a known beta distribution to update the value of ρ_*s*_. Genovo sets ρ_*s*_ to the mode of the Beta distribution where ${O}_{s}=\sum _{k=1}^{{N}_{s}}\vert {O}_{k}\vert $.

**Figure 4:**
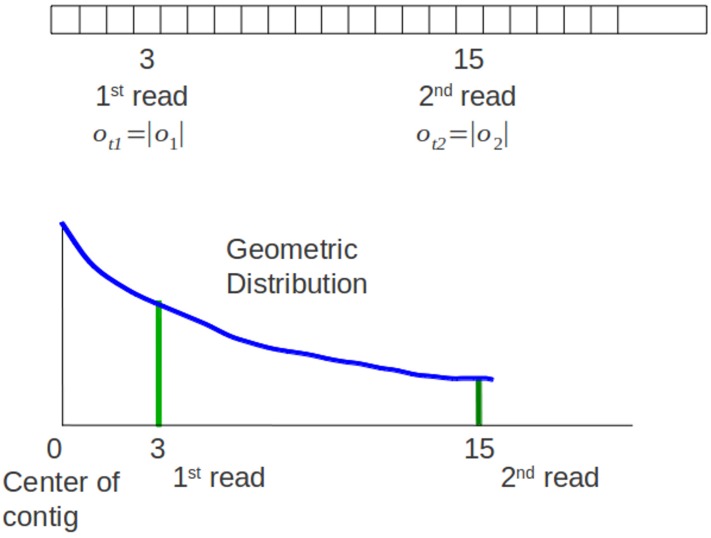
Original sampling process. The 1^st^ read and 2^nd^ read are mapped in a contig. There is a graph of the symmetric geometric distribution of the reads in the positive integer side. The center of the distribution is the center of contig (0).

**Figure 5:**
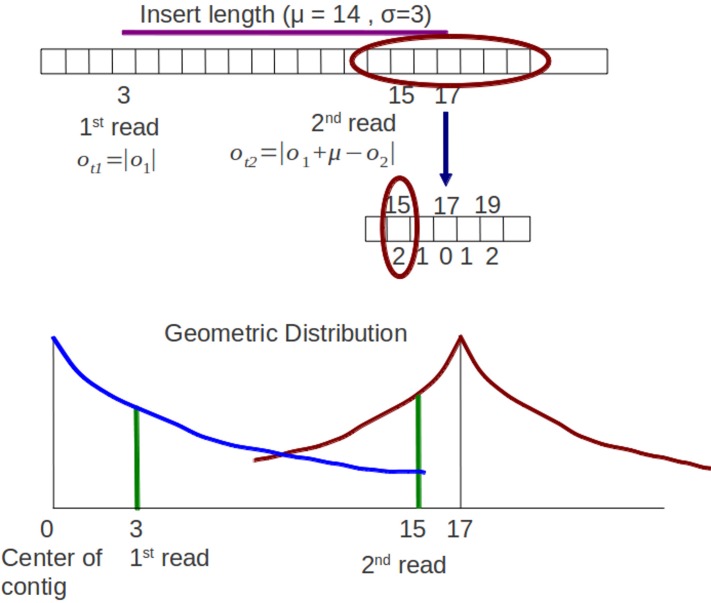
Modified sampling process. The 1^st^ read and 2^nd^ read are mapped in a contig. Insert length distribution is (μ, δ) = (14, 3). The distribution of the 1^st^ read and 2^nd^ read have different centers. The center of the 2^nd^ read incorporates the insert length parameter, the center is 3 (the offset of 1^st^ read) + 14 (insert length) = 17.

For paired end reads, the sampling process of offset should take into account the insert length parameter. Xgenovo uses the relative distance of a read to its mate incorporating the insert length. In the illustration shown in Fig. 4, there are paired end reads with insert length distribution (μ, δ) = (14, 3). The 1^st^ read is mapped in the offset 3 and the 2^nd^ read is mapped in the offset 15. Genovo uses the absolute value of the offset as the number of trials, hence the number of trials for the 1^st^ read is 3 and for the 2^nd^ read is 15. From the illustration, we can see that the 2^nd^ read sampled in the appropriate location has lower probability than in the location which is close to the center of the contig. It happens because the center of the symmetric geometric distribution for the 2^nd^ read is the center of the contig and doesn’t take into consideration the insert length parameter. While in Xgenovo, the number of trials for the 1^st^ read is the same as Genovo (3) yet relative distance is used for the 2^nd^ read. The relative distance is defined by |*o*_1_ + μ−*o*_2_|. Xgenovo utilizes the insert length parameter to determine the center of the distribution of the 2^nd^ read. Therefore the 2^nd^ read sampled in the appropriate location has higher probability. In the illustration shown in Fig. 5, the number of trials is *o*_*t*2_ = |3 + 14−15| = 2. The formula of symmetric geometric distribution for the 1^st^ read is same with Genovo shown in (13), while the distribution for the 2^nd^ read is shown in (14).

(13) \begin{eqnarray*} \displaystyle G({o}_{1}\vert {\rho }_{1\mathrm{s}})=\left\{\begin{array}{@{}ll@{}} \displaystyle 0.5(1-{\rho }_{1\mathrm{s}})^{\vert {o}_{1}\vert }{\rho }_{1\mathrm{s}}&\displaystyle {o}_{1}\not = 0\\ \displaystyle {\rho }_{1\mathrm{s}}&\displaystyle {o}_{1}=0 \end{array}\right.&&\displaystyle \end{eqnarray*}

(14) \begin{eqnarray*} \displaystyle G({o}_{2\mathrm{s}}\vert {o}_{1},{o}_{2},{\rho }_{2\mathrm{s}})=\left\{\begin{array}{@{}ll@{}} \displaystyle 0.5(1-{\rho }_{2\mathrm{s}})^{\vert {o}_{t 2}\vert }{\rho }_{2\mathrm{s}}&\displaystyle {o}_{t 2}\not = 0\\ \displaystyle {\rho }_{2\mathrm{s}}&\displaystyle {o}_{t 2}=0 \end{array}\right.\quad \text{where }{o}_{t 2}=\vert {o}_{1}+\mu -{o}_{2}\vert &&\displaystyle \end{eqnarray*}

There is a possibility that the 2^nd^ read is not sampled in the same contig with the 1^st^ read. For this case, both the 1^st^ read and the 2^nd^ read are considered as 1^st^ read (single read). There are two ρ_*s*_, ρ_1*s*_ for the 1^st^ read and ρ_2*s*_ for the 2^nd^ read. Both are updated using known Beta $(1+{N}_{1\mathrm{s}},1+\beta +{O}_{1\mathrm{s}})=\frac{{N}_{1\mathrm{s}}}{{N}_{1\mathrm{s}}+\beta +{O}_{1\mathrm{s}}}$ distributions. The ρ_1*s*_ is updated by the mode of distribution Beta where ${O}_{1\mathrm{s}}=\sum _{k=1}^{{N}_{1\mathrm{s}}}\vert {o}_{1\mathrm{k}}\vert $. The ρ_2*s*_ is updated by the mode of $(1+{N}_{2\mathrm{s}},1+\beta +{O}_{t 2 s})=\frac{{N}_{2\mathrm{s}}}{{N}_{2\mathrm{s}}+\beta +{O}_{t 2 s}}{O}_{t 2 s}=\sum _{k=1}^{{N}_{2\mathrm{s}}}\vert {o}_{t 2 k}\vert $ distribution Beta where *N*_1*s*_ is the number of the 1^st^ read or single read (read which is not in the same contig with its mate) in contig *s*, *o*_1_ is the offset of a read, *N*_2*s*_ is the number of the 2^nd^ read in contig *s* and *o*_*t*2_ is the number of trial for 2^nd^ read. By using this relative distance, reads sampled in the appropriate location in a contig has a higher probability of model so that a contig produced is correct compared to using default distance in Genovo.

#### Likelihood

The probability distribution in CRP and sampling process are changed so that the likelihood of the model also changes. Like Genovo, the likelihood of our model also consists of 4 components, shown in (4). The likelihood of the alignments log *p*(***x***, ***y***|***s***, ***o***, ***b***) and the likelihood for generating (uniformly) each contig letter log *p*(***b***) are the same as Genovo’s. While the differences are for the likelihood of contigs, shown in (15) and the likelihood of offsets, shown in (16)–(18).

**Figure 6:**
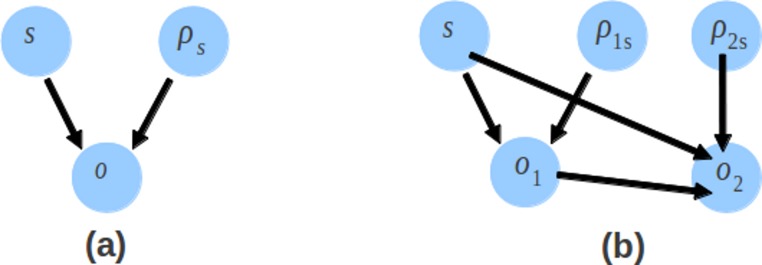
The directed graphical model representing the likelihood of offsets. The likelihood of offsets for the original Genovo (A) in the left side with 3 nodes, and the extended Genovo (B) in the right side with 5 nodes.

(15) \begin{eqnarray*} \displaystyle \log \hspace{0.167em} p(s)=S\log (\alpha )+\sum _{i=1}^{S}\log \hspace{0.167em} \Gamma ({N}_{s})+\log \hspace{0.167em} \Gamma (\alpha )-\log \hspace{0.167em} \Gamma (N+\alpha )+{N}_{2\mathrm{s}}\log (\beta )&&\displaystyle \end{eqnarray*}

(16) \begin{eqnarray*} \displaystyle \log \hspace{0.167em} p(o\vert s,{\rho }_{1\mathrm{s}},{\rho }_{2\mathrm{s}})=\log \hspace{0.167em} p({o}_{1}\vert s,{\rho }_{1\mathrm{s}})+\log \hspace{0.167em} p({o}_{2}\vert s,{\rho }_{2\mathrm{s}})&&\displaystyle \end{eqnarray*}

(17) \begin{eqnarray*} \displaystyle \log \hspace{0.167em} p({o}_{1}\vert s,{\rho }_{1\mathrm{s}})=\sum _{i=1}^{S}[{O}_{1\mathrm{s}}\log (1-{\rho }_{1\mathrm{s}})+{N}_{1\mathrm{s}}\log \hspace{0.167em} {\rho }_{1\mathrm{s}}+{N}_{1\mathrm{s}}\log \hspace{0.167em} 0.5]&&\displaystyle \end{eqnarray*}

(18) \begin{eqnarray*} \displaystyle \log \hspace{0.167em} p({o}_{2}\vert s,{o}_{1},{\rho }_{2\mathrm{s}})=\sum _{i=1}^{S}[{O}_{t 2 s}\log (1-{\rho }_{2\mathrm{s}})+{N}_{2\mathrm{s}}\log \hspace{0.167em} {\rho }_{2\mathrm{s}}+{N}_{2\mathrm{s}}\log \hspace{0.167em} 0.5]&&\displaystyle \end{eqnarray*}

where *S* is the number of contigs, *N_s_* is the number of read in contig *s*, ${O}_{1\mathrm{s}}=\sum _{k=1}^{{N}_{1\mathrm{s}}}\vert {o}_{1\mathrm{k}}\vert {O}_{t 2 s}=\sum _{k=1}^{{N}_{2\mathrm{s}}}\vert {o}_{t 2 k}\vert ,{N}_{1 s}$ is the number of 1^st^ read or single end read in contig *s* and *N*_2*s*_ is the number of 2^nd^ read in contig *s*. There is an additional component for the likelihood of contigs which takes into account the bonus parameter and the number of 2^nd^ reads. The likelihood of offset consists of the likelihood of the offset of the 1^st^ read and the likelihood of the offset of the 2^nd^ read. The directed graphical model representing the likelihood of offsets is shown in Fig. 6. Genovo has 3 variables (*o*, ρ_*s*_ and *s*), the probability of offset (*o*) given ρ_*s*_ and *s*. Xgenovo has 5 variables (*o*_1_, *o*_2_, ρ_1*s*_, ρ_2*s*_ and *s*), the probability of the offset of the 1^st^ read (*o*_1_) given ρ_1*s*_ and *s*, the probability of the offset of the 2^nd^ read (*o*_2_) given *o*_1_, ρ_2*s*_ and *s*. Like Genovo, Xgenovo also outputs the assembly that achieved the highest likelihood thus far.

## Results and Discussion

We used Metasim (Richter et al., 2008) to generate simulated metagenomic datasets. The read length was set at 250 bp and used the default 454 sequencing noise provided by Metasim. The insert length distribution (μ, δ) is (3000, 200). We generated 50,000 pairs of reads for each dataset which is twice the size of the simulated dataset used in Genovo’s paper. To evaluate the performances of metagenomic assemblers comprehensively, we applied two kinds of species coverage (abundance) distribution for the dataset, uniform distribution and log-normal distribution. Uniform distribution means that each species in the dataset has the same probability to exist, or it can be said that each species has same abundance value or similar to each other. Second, we applied species abundance following log-normal distribution. The log-normal distribution appropriately describes the microbial abundance distributions (Unterseher et al., 2011). We generated simulated metagenomic datasets from simple to complex datasets. The complexity of dataset is based on the number of genomes in the dataset (Mende et al., 2012). For log-normal distribution, first we generated the simplest dataset consisting of 13 viruses which is the same complexity with a simulated dataset used in Genovo’s paper, with the lowest coverage = 7.42x, the highest coverage = 188.93x, as LC and HC respectively, then the 2^nd^ dataset consists of 17 viruses (LC = 10.82x, HC = 363.18x), the 3^rd^ dataset consists of 30 viruses (LC = 6.64x, HC = 708.79x) and the 4^th^ dataset consists of 35 viruses (LC = 10.59x, HC = 492.23x). For uniform distribution, we generated 4 simulated metagenomic datasets which contain 35 viruses with the same coverage for each species. In the 1^st^ dataset each species has 30 times coverage of the genome sequences; in the 2^nd^ dataset each species has 40; in the 3^rd^ dataset each species has 50 and in the 4^th^ dataset each species has 60.

We compared the performance of Xgenovo with the naive use of the original Genovo and MAP. In the MAP’s paper, they used datasets consisting of 113 species therefore, to compare the performance between Xgenovo and MAP more rigorously, we generated very complex datasets consisting of 50 viruses, 60 viruses, 90 viruses and 115 viruses, both for log-normal distribution, 50 viruses (LC = 9.10x, HC = 427.04x), 60 viruses (LC = 3.95x, HC = 648.49x), 90 viruses (LC = 8.46x, HC = 831.79x), 115 viruses (LC = 10.52x, HC = 1986.55x) and for uniform distribution, with the same coverage: 50 viruses (50x), 60 viruses (50x), 90 viruses (40x) and 115 viruses (55x). In total, we generated 16 simulated datasets. The complete descriptions of all datasets are provided in http://xgenovo.dna.bio.keio.ac.jp.

In order to evaluate the assembly capacity, we used four measurements: N50, total length of contig, maximum length of contig and the number of contigs. To evaluate the assembly quality we used two measurements: cover rate and chimera rate. We were also concerned with the computational cost, CPU time and required memory. N50 is a standard statistical measure evaluating the assembly performance which indicates the largest value *y* such that at least 50% of the genome is covered by contigs of length of ≥*y*. We follow Namiki et al. (2012) to measure the cover rate and chimera rate. The cover rate of genome X is defined as the ratio of the total length of contigs which are best aligned to genome X divided by the length of genome X, shown in (19), where *C_i_* is the length of contig *i* which is best aligned to genome *A*.

(19) \begin{eqnarray*} \displaystyle \text{Cover rate of }A=\frac{(\sum \vert C_{i}\vert )}{\vert A\vert }&&\displaystyle \end{eqnarray*}

To determine whether a contig is chimeric or not: first, the best hit alignments between a contig and the set of input reference genomes using BLAST is calculated; second, if a contig has more than two subsequences that are aligned to different genomes, and those subsequences are longer than 1% of the contig length, the contig is determined to be chimeric.

We compared the performance of Xgenovo with the naive use of the original Genovo and MAP. Genovo set the parameter α = 2^35^, the best parameter value to assemble. To know the performance of Xgenovo, we used combinations of parameters between α and β (bonus). The combinations were α = 2^35^ and β = 0.1α, 0.3α, 0.5α. We ran both Xgenovo and Genovo for 200 iterations which reaches convergence. We used the default setting for MAP. All computations were executed with Intel(R) Xeon(R) E5540 processors (2.53 GHz) and 48 GB physical memory.

### Experiments on different numbers of species with coverage following log-normal distribution

**Figure 7:**
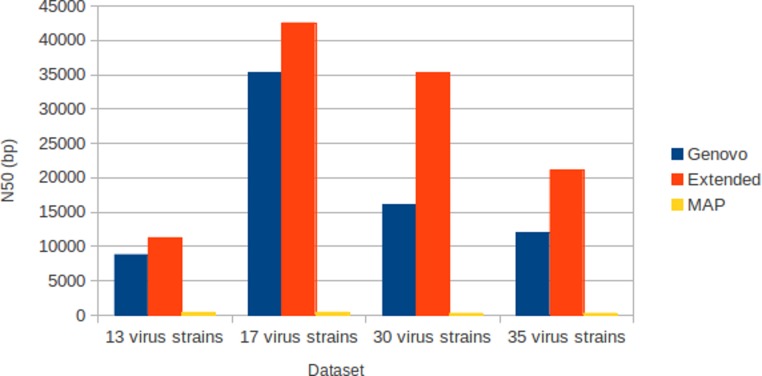
N50 for experiments on different numbers of species with coverage following log-normal distribution. Xgenovo generated the highest N50. The *x*-axis is the name of the dataset and the *y* axis is the N50 (bp).

The results for experiments on different numbers of species with coverage following log-normal distribution were shown in Table 1. The results were the best performances of parameter combinations between α and β. Xgenovo generated the highest N50 for all datasets, shown in Fig. 7. Compared to the original Genovo, Xgenovo increased N50 by 28.1% (2473 bp) for the dataset with 13 viruses, increased N50 by 20.3% (7202 bp) for the dataset with 17 viruses, increased N50 by 119.5% (19213 bp) for the dataset with 30 viruses and increased N50 by 75.0% (9112 bp) for the dataset with 35 viruses. Xgenovo assembled significantly longer N50 than other assemblers. Xgenovo generated similar values for the total length of contigs and the number of contigs. Xgenovo increased the maximum length of contig, except for a dataset with 13 viruses, Xgenovo generated a similar value of maximum length (Genovo = 21101 bp, Xgenovo = 21098 bp). MAP generated the lowest assembly capacity.

**Table 1:** Experiments on different numbers of species with coverage following log-normal distribution.

Metagenome datasets	Genovo	Xgenovo	MAP
**13 viruses**		***β* = 0.3 *α***	
N50 (bp)	8790	11263	399
Total length (bp)	111777	112059	1033790
Max length (bp)/# Contig	21101/13	21098/12	749/2895
Cover rate (%)/Chimera rate (%)	91.15/0	91.15/0	74.05/0
CPU time (s)/Memory (GB)	2365/0.99	2393/1.009	10481/6.053
**17 viruses**		***β* = 0.3 *α***	
N50 (bp)	35308	42510	417
Total length (bp)	382890	382183	1348420
Max length (bp)/# Contig	145725/22	168835/17	872/3540
Cover rate (%)/Chimera rate (%)	96.87/0	96.87/0	33.59/0
CPU Time (s)/Memory (GB)	7423/1.301	5246/1.242	4213/6.445
**30 viruses**		***β* = 0.3 *α***	
N50 (bp)	16068	35281	258
Total length (bp)	470033	468819	305091
Max length (bp)/# Contig	84444/93	168713/80	897/1050
Cover rate (%)/Chimera rate (%)	95.58/0	95.57/0	37.59/0
CPU time (s)/Memory (GB)	9067/1.372	4653/1.312	28240/10.637
**35 viruses**		***β* = 0.3 *α***	
N50 (bp)	11993	21105	259
Total length (bp)	530538	535103	321620
Max length (bp)/# Contig	155979/61	168736/74	722/1064
Cover rate (%)/Chimera rate (%)	97.01	97	39.82
CPU time (s)/Memory (GB)	25504/1.443	9240/1.411	17131/7.598

**Figure 8:**
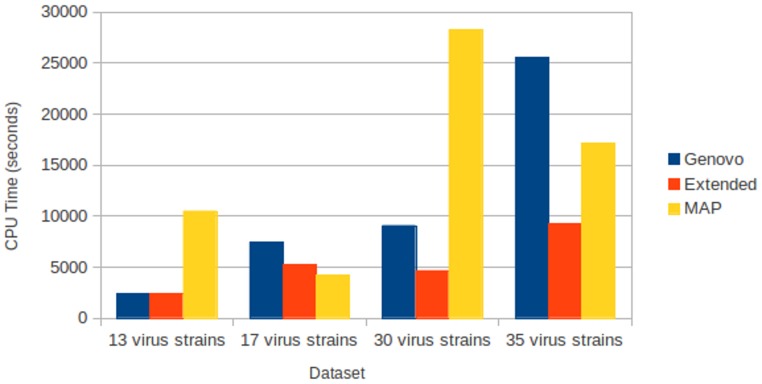
CPU time required for experiments on different numbers of species with coverage following log-normal distribution. The *x*-axis is the name of the dataset and the *y*-axis is the CPU time required (seconds).

All assemblers generated no chimera contig (chimera rate = 0%). Xgenovo generated similar cover rate with the original Genovo, while MAP generated the lowest cover rate. Figure 8 shows the CPU time required. Compared to the original Genovo, Xgenovo decreased CPU time by 29.3% (2177 s) for the dataset with 17 viruses, by 48.6% (4414 s) for the dataset with 30 viruses and by 63.7% (16264 s) for the dataset with 35 viruses.

### Experiments on different coverage with uniform distribution

**Figure 9:**
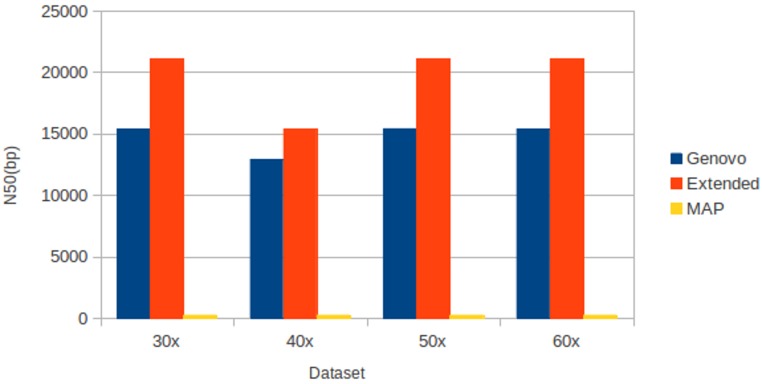
N50 for experiments on different coverages with uniform distribution. Xgenovo generated the highest N50. The *x* axis is the name of the dataset and the *y* axis is the N50 (bp).

The results for experiments on different coverage with uniform distribution were shown in Table 2. Like datasets of log-normal distribution, Xgenovo generated higher N50 than Genovo and MAP for all datasets, shown in Fig. 9. Compared to the original Genovo, Xgenovo increased N50 by: 36.93% (5694 bp) for datasets with the same coverage 30x; 19.04% (2464 bp) for datasets with the same coverage 40x; 36.77% (5674 bp) for datasets with the same coverage 50x and 36.83% (5676 bp) for datasets with the same coverage 60x. Xgenovo generated similar values for the total length of contigs and the number of contigs. Xgenovo increased maximum length of contig; except for datasets with the same coverage 30x, Xgenovo generated similar values of maximum length (Genovo = 167396 bp, Xgenovo = 163305 bp). Like with log-normal distribution, MAP generated the lowest assembly capacity.

**Table 2:** Experiments on different coverage with uniform distribution.

Metagenome datasets	Genovo	Xgenovo	MAP
**Same coverage 30x**		***β* = 0.3 *α***	
N50 (bp)	15415	21109	256
Total length (bp)	533648	534797	210539
Max length (bp)/# Contig	167396/35	163305/36	482/814
Cover rate (%)/Chimera rate (%)	97.57/0	97.57/0	28.19/0
CPU time (s)/Memory (GB)	3597/1.457	3149/1.459	7128/4.818
**Same coverage 40x**		***β* = 0.1 *α***	
N50 (bp)	12937	15401	256
Total length (bp)	535585	534993	212998
Max length (bp)/# Contig	84733/40	160954/37	480/824
Cover rate (%)/Chimera rate (%)	97.57/0	97.58/0	27.72/0
CPU time (s)/Memory (GB)	7158/1.479	5539/1.439	7679/4.862
**Same coverage 50x**		***β* = 0.1 *α***	
N50 (bp)	15429	21103	256
Total length (bp)	534353	535576	212832
Max length (bp)/# Contig	157303/36	169161/35	483/823
Cover rate (%)/Chimera rate (%)	97.56/0	97.57/0	27.87/0
CPU time (s)/Memory (GB)	3267/1.439	3580/1.441	7173/4.818
**Same coverage 60x**		***β* = 0.1 *α***	
N50 (bp)	15410	21086	256
Total length (bp)	534771	535626	218786
Max length (bp)/# Contig	146185/37	169024/36	477/849
Cover rate (%)/Chimera rate (%)	97.58/0	97.58/0	29.27/0
CPU time (s)/Memory (GB)	6069/1.44	6072/1.418	7378/4.812

**Figure 10:**
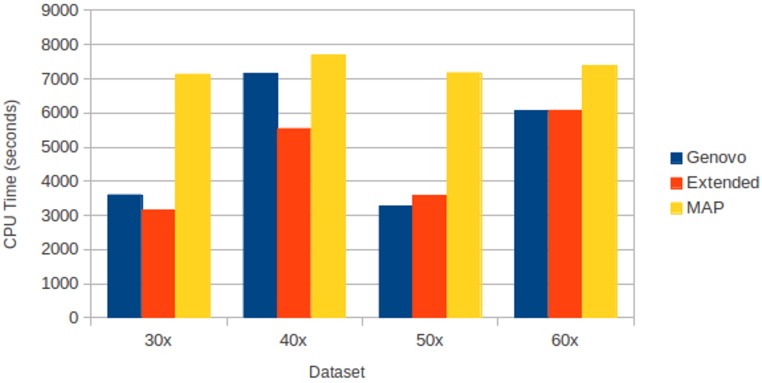
CPU time required for experiments on different coverages with uniform distribution. The *x*-axis is the name of the dataset and the *y*-axis is the CPU time required (seconds).

All assemblers generated 0 for chimera rate. Xgenovo generated a similar cover rate with the original Genovo, while MAP generated the lowest cover rate. Figure 10 shows the CPU time required. Compared to the original Genovo, Xgenovo decreased CPU time by 12.45% (448 s) for datasets with the same coverage 30x and by 22.60% (1619 s) for datasets with the same coverage 40x.

### Experiments on very complex datasets

To compare the performance between Xgenovo and MAP more rigorously, we generated very complex datasets consisting of 50 viruses, 60 viruses, 90 viruses and 115 viruses, both for log-normal and uniform distribution. The results were shown in Table 3. Xgenovo generated much higher N50 than MAP for all datasets. Xgenovo generated from 7 times N50 than MAP (for the dataset with 115 viruses with log-normal distribution) until 337 times N50 than MAP (for the dataset of 50 viruses with uniform distribution). For the dataset with 115 viruses with log-normal distribution, MAP generated N50 = 318 bp while Xgenovo generated N50 = 2457 bp. For the dataset with 50 viruses with uniform distribution, MAP generated N50 = 288 bp while Xgenovo generated N50 = 97184 bp. MAP generated a lower chimera rate than Xgenovo but MAP generated very low N50, no more than 392 bp for all datasets while the length of the read is 250 bp. The results showed that Xgenovo outperforms MAP even for very complex datasets.

**Table 3:** Experiments on very complex datasets.

Metagenome datasets	Xgenovo	MAP
**50 Viruses log-normal**	***β* = 0.5 *α***	
N50 (bp)	28903	278
Total length (bp)	1761195	5396779
Max length (bp)/# Contig	110113/502	1479/17966
Cover rate (%)/Chimera rate (%)	94.84/0.93	92.75/0
CPU time (s)/Memory (GB)	39738/2.566	52449/7.906
**50 Viruses uniform**	***β* = 0.5 *α***	
N50 (bp)	97184	288
Total length (bp)	1794418	5293906
Max length (bp)/# Contig	194028/123	1123/17519
Cover rate (%)/Chimera rate (%)	96.99/6.07	97.1/0
CPU time (s)/Memory (GB)	41324/2.761	25193/4.147
**60 Viruses log-normal**	***β* = 0.1 *α***	
N50 (bp)	10636	293
Total length (bp)	2362232	5478753
Max length (bp)/# Contig	108811/1120	1633/17258
Cover rate (%)/Chimera rate (%)	92.38/1.19	87.59/0
CPU time (s)/Memory (GB)	43206/3.228	16502/3.625
**60 Viruses uniform**	***β* = 0.3 *α***	
N50 (bp)	25099	301
Total length (bp)	2840986	5283695
Max length (bp)/# Contig	106273/346	1476/16628
Cover rate (%)/Chimera rate (%)	96.77/1.06	24.44/0.09
CPU time (s)/Memory (GB)	43786/3.618	14478/3.232
**90 Viruses log-normal**	***β* = 0.3 *α***	
N50 (bp)	2916	303
Total length (bp)	3003232	5622001
Max length (bp)/# Contig	126010/2640	1864/16890
Cover rate (%)/Chimera rate (%)	85.14/0.63	77.52/0.02
CPU time (s)/Memory (GB)	60162/ 4.27	21104/3.75
**90 Viruses uniform**	***β* = 0.5 *α***	
N50 (bp)	6480	362
Total length (bp)	3631520	5728435
Max length (bp)/# Contig	25751/1507	1785/15562
Cover rate (%)/Chimera rate (%)	96.03/1.39	90.85/0.01
CPU time (s)/Memory (GB)	56635/4.27	11656/2.76
**115 Viruses log-normal**	***β* = 0.5 *α***	
N50 (bp)	2457	318
Total length (bp)	3437804	5689659
Max length (bp)/# Contig	128459/3410	1806/16620
Cover rate (%)/Chimera rate (%)	81.33/0.84	73.93/0.01
CPU time (s)/Memory (GB)	68789/4.96	10480/ 3.42
**115 Viruses uniform**	***β* = 0.1 *α***	
N50 (bp)	4264	392
Total length (bp)	4518801	6269732
Max length (bp)/# Contig	27417/2720	1956/16104
Cover rate (%)/Chimera rate (%)	96.07/0.59	88.07/0.02
CPU time (s)/Memory (GB)	72487/5.29	8137/2.23

### Discussion

For datasets of log-normal and uniform datasets, compared to Genovo, Xgenovo successfully assembled longer N50. Longer contigs can help extract more information from the reads leading to the discovery of more genes and better functional annotation (Meyer et al., 2009). When the N50 score is longer, more complete protein-coding genes are predicted (Namiki et al., 2012). Xgenovo also successfully generated higher maximum length of contig in most datasets. These results mean that Xgenovo can increase the assembly capacity. Although it increased the assembly capacity, Xgenovo maintained assembly quality by generating a competitive cover rate and chimera rate value. Compared to MAP, Xgenovo also generated a much higher N50. MAP generated low value for both assembly capacity and quality. For metagenomic datasets which are at low taxonomic level, the genomes become more similar and share more reads with each other. MAP uses an improved OLC (Overlap/Layout/Consensus) strategy to integrate mate pair information which treats a read as a node, therefore the more similar the genomes, the more complex the graph. It might be a reason why MAP generated low performance.

Aside from the improved assembly performance, Xgenovo demonstrated the potential to decrease the computational cost. As explained in the previous section, Genovo uses iterative procedures to discover appropriate assemblies. The main iterative procedure is read mapping. This procedure updates the position of the read in the coordinate system of contigs and offsets. This procedure samples the contig of a read utilizing CRP and samples the location of read in the contig utilizing symmetric geometric distribution. This procedure requires the highest computational cost of procedures in Genovo. A read will be resampled if its mapping location in the contig contains some problematic spots. A problematic spot is defined as a spot having supported reads ≤ 2, a spot in the edge of a contig, or a spot which doesn’t have a supported read before or after it. If a read doesn’t have any problematic spots, the read will not be resampled. In Xgenovo, CRP and the symmetric geometric distribution are modified so that a read sampled in the appropriate location has higher probability which means that a read will be mapped in the correct location. If a read is mapped in the correct location, it contains fewer problematic spots and doesn’t need to be resampled. That is why it’s possible for Xgenovo to decrease the computational time in the same number of iterations.

## Conclusion

We successfully extended Genovo by incorporating paired-end information so that it generates higher quality assemblies with paired end reads by modifying Genovo in determining the location of a read in the coordinate system of the contig and the offset (the beginning of the read). Unlike other assemblers (Koren, Treangen & Pop, 2011; Li et al., 2010; Namiki et al., 2012; Peng et al., 2012; Zerbino & Birney, 2008; Zerbino et al., 2009) which use paired end information to generate scaffolds, we attempted to increase the assembly performance without the aim of generating scaffold but attempted to map reads to the contigs in the correct location. Xgenovo successfully generated longer N50 than the original Genovo and the recently proposed matagenome assembler for 454 reads, MAP while maintaining the assembly quality for simulated metagenomic datasets with species coverage following uniform and log-normal distribution even for very complex dataset. Xgenovo also demonstrated the potential to decrease the computational cost. It means that our strategy worked well.

Genovo is the only metagenomic assembler that uses a generative probabilistic model. Unlike the other methods, Genovo assembles all reads without discarding any reads. This strategy avoids filtering out any low coverage genomes, hence hopefully is able to extract more information from metagenomic data in order to generate better assembly results. The consequence is high computational cost. We have improved Genovo by incorporating paired end information and demonstrate that it can reduce computational cost. Short reads, for example Illumina reads, have been gaining popularity, even for metagenomic studies (Hiatt et al., 2010). We are going to continue our research and extend our method for short read data in order to generate high assembly accuracy and capacity with reliable computational cost. Current metagenomic assemblers for short read data (Metavelvet, MetaIDBA and IDBA-UD) use the De Bruijn graph approach. Therefore, the implementation of a probabilistic model for short read data with high assembly performances and consistent computational cost is a potential area of research.

## Additional Information and Declarations

### Data Deposition

The following information was supplied regarding the deposition of related data: http://xgenovo.dna.bio.keio.ac.jp/download.
